# Velocity‐Dependent Transitions in Forward and Backward Running Mechanics in Female International Rugby Players

**DOI:** 10.1002/ejsc.70255

**Published:** 2026-09-11

**Authors:** Tayah R. Brennan, Jonathon Weakley, Grant Duthie, Mark Moresi, Nicholas Cowley, Kade Silverthorne, Isabella Hebron, Laura Starc, Nirav Maniar, Mark W. Creaby

**Affiliations:** ^1^ School of Behavioural and Health Sciences, Faculty of Health Sciences Australian Catholic University Brisbane Australia; ^2^ Sports Performance, Recovery, Injury, and New Technologies (SPRINT) Research Centre Mary Mackillop Institute for Health Research Australian Catholic University Brisbane Australia; ^3^ Carnegie Applied Rugby Research (CARR) Centre Carnegie School of Sport Leeds Beckett University Leeds UK; ^4^ Rugby Australia National Rugby Training Centre Brisbane QLD Australia; ^5^ Global Sport and Movement Collaborative University of Newcastle Newcastle New South Wales Australia; ^6^ School of Biomedical Sciences and Pharmacy College of Health, Medicine, and Wellbeing University of Newcastle Newcastle Australia

**Keywords:** athletic performance, female athletes, running biomechanics, sprint mechanics, stride length–frequency relationship

## Abstract

This study aimed to examine the relationship between spatiotemporal variables and velocity in forward and backward running and identify if, and where, distinct transitions in these relationships occur in female international representative rugby sevens athletes. Sixteen female international representative rugby sevens athletes completed forward and backward overground running from 40% to 100% of maximum velocity. Spatiotemporal variables were derived from ground embedded force plates and three‐dimensional motion analysis. A segmented mixed modelling approach was used to model the relationship between spatiotemporal variables and running velocity. At velocities up to 5.95 and 3.27 m·s^−1^ in forward and backward running, respectively, increases in ground contact distance, swing distance, and hence, stride length were observed. In forward running, our analyses revealed a distinct plateau in stride length at velocities above 5.95 m·s^−1^, with a rapid rise in stride frequency thereafter. Backward running exhibited a similar, but distinct strategy at higher velocities; the increase in stride length became more conservative above 3.27 m·s^−1^, whereas stride frequency became more prominent. Reductions in ground contact time and swing time accompanied the increase in stride frequency. Athletes displayed a greater reliance on increasing stride length at lower velocities, but its contribution declined as velocity increased. Thus, a change in strategy to that of increasing stride frequency was required. Forward running velocity may be enhanced by promoting rapid leg turnover during the swing‐phases. Conversely, a focus on both force application during ground contact and rapid leg turnover during the swing‐phases would appear best to enhance backward running velocity.

## Highlights

- Female rugby sevens athletes showed a greater reliance on increasing stride length at velocities below 5.95 and 3.27 m·s^−1^ (∼75% and ∼65%*V*_max_) in forward and backward running, respectively.
- Stride frequency became the primary driver of speeds above 5.95 and 3.27 m·s^−1^ (∼75% and ∼65%*V*_max_) in forward and backward running, respectively. A focus on improving stride frequency would likely be the best approach to improve top‐speed. Exercises such as overspeed and resisted running encourage higher stride frequencies and thus may be beneficial in this regard.
- Maximal backward running may demand increases in both stride length and stride frequency. We suggest that working on improving both stride length and stride frequency would likely be the best approach to improve top backward running speed. A combination of drills to maximise force application during foot‐ground contact (e.g., plyometrics) and rapid leg turnover during the swing‐phases (e.g., overspeed and resisted running) may be applied.

## 1 Introduction

Running performance in rugby is defined by repeated bouts of high‐speed forward running and rapid defensive retreat (Bicudo et al. 2025; Hogarth et al. 2016). Although forward running constitutes most sport locomotion, athletes will also run in a backwards direction when required to maintain visual view of opponents during phases of defensive retreat (Sirotic et al. 2011). Therefore, backward running is essential for critical defensive actions, and whilst its importance will vary across different sports, it is notable that rugby league players run an average of 3.6–5.4 m in a backwards direction after performing a tackle (Sirotic et al. 2011). Equivalently, forward running underpins line speed and overall game involvement (Hogarth et al. 2016). Taken together, the capacity for high‐speed movement in both forward and backward directions is critical for gaining a tactical advantage. Accordingly, understanding the mechanical determinants of forward and backward running velocity will enable more targeted interventions to enhance rugby‐specific performance.

Fundamentally, running velocity is driven by alterations in stride length and stride frequency (Mero et al. 1992; Luhtanen and Komi 1978). In forward running, numerous studies propose that increases in stride length reach a functional limit as velocity increases, with stride frequency becoming increasingly influential thereafter (Willer et al. 2014; Dorn et al. 2012; Weyand et al. 2000). Indeed, at higher forward running velocities, ground contact times become progressively shorter, reducing the time available over which force can be applied, and thereby constraining the athlete's ability to project the centre of mass forward and upward (Schache et al. 2014); this projection is what typically results in longer strides (Hunter et al. 2004). Conversely, reductions in swing time at higher forward running velocities suggests that athletes reposition their legs more rapidly, which facilitates a higher stride frequency (Willer et al. 2014; Weyand et al. 2000). Together, this implies a distinct shift in movement strategy as athletes approach maximal speed, that also reflects coordinated changes in stance (i.e., ground contact) and swing phase mechanics (Hay 1994).

Despite the clear contribution of backward running in rugby, the mechanical determinants of backward running remain poorly characterised in overground, sport‐specific contexts. Existing biomechanical studies of backward running have generally examined a single constant velocity (Devita and Stribling 1991; Threlkeld et al. 1989; Flynn et al. 1994) or relied on treadmill protocols (Weyand et al. 2010). It should be noted that, in forward locomotion, the strategies used to achieve faster running velocities while running on a treadmill differ from those used in overground running (Bailey et al. 2017). Thus, the determinants of backward running performance in game‐relevant overground conditions are unknown. Importantly, backward running is mechanically distinct, involving different muscle activations, force characteristics, and joint loading demands (Flynn and Soutas‐Little 1993, 1995; Threlkeld et al. 1989), which may influence how stride length and stride frequency are generated and constrained. In addition, altered visual feedback in backward running increases the demands on spatial awareness and anticipatory control (Duysens et al. 2014; Mehdizadeh et al. 2015), potentially leading to more conservative centre of mass displacement, longer ground contact times, or modified swing mechanics. As a result, spatiotemporal strategies likely differ from forward running, suggesting direction‐specific interventions would be required.

Understanding the spatiotemporal determinants of velocity in both forward and backward running is especially important for female rugby sevens players. Rugby sevens is characterised by high relative running demands. Reviews of rugby sevens match demands indicate that players cover large relative running distances and may experience greater running demands than athletes in other rugby codes and field‐based team sports (Henderson et al. 2018; Hogarth et al. 2016). Importantly, female athletes have been shown to exhibit distinct mechanical responses compared to their male counterparts (Haugen et al. 2020; Debaere et al. 2013), suggesting that performance determinants differ between sexes. A key difference emerges during the transition to top speed, particularly in how stride length and stride frequency are regulated as velocity increases. Female athletes show a strong inverse relationship between these variables, whereby, increases in stride frequency are accompanied by reductions in stride length (Debaere et al. 2013). In contrast, males are able to increase stride frequency without substantially compromising stride length (Debaere et al. 2013). Consequently, these sex‐specific coordination strategies are likely to play a key role in overall high‐speed running performance. Without sex‐specific data, it is difficult to determine whether current training interventions adequately address female running mechanics and the constraints shaping them. Compounding this issue, compared to trained sprinters, athletes in team sports exhibit lower reactive‐strength, stiffness regulation, and subsequently, lower maximum velocities (Douglas et al. 2020), meaning their running mechanics cannot be accurately inferred from sprinter‐based evidence. To design evidence‐based training interventions that genuinely reflect the biomechanics of female rugby sevens athletes, we must examine the spatiotemporal strategies that these athletes use.

This study examines the spatiotemporal determinants of velocity in forward and backward running to provide foundational insight into the mechanical strategies underpinning performance in female international representative rugby sevens athletes. We employ a segmented mixed modelling approach to; (I) examine the relationship between spatiotemporal variables and running velocity, and (II) identify distinct transitions in this relationship. We hypothesise that distinct velocity‐dependent transitions will occur, whereby the spatiotemporal strategy used by female rugby sevens athletes in forward and backward running will differ when running at lower or higher velocities. This transition is expected to reflect coordinated changes in ground contact and swing phase mechanics.

## 2 Materials & Methods

### 2.1 Study Design & Participants

Sixteen female rugby sevens athletes (age: 19–24, 22 ± 2 years; height: 169 ± 4 cm; body mass: 72 ± 6 kg) from the Australian Women's Rugby Sevens national squad were recruited for this observational study. This sample included five Tier 4 (Elite/International) and 11 Tier 5 (World Class) athletes, with Tier five identified by achieving Olympic or world medallist status (McKay et al. 2022). Eligibility criteria required that participants were over 18 years of age, currently injury free, and cleared for full training by the team medical staff. Participants provided written and informed consent prior to participation. The study was approved by an Institutional Human Research Ethics Committee (Ethics number: 2023‐3814H) and reported here in accordance with the STROBE guidelines (von Elm et al. 2008).

### 2.2 Procedures

All running bouts were performed on a 60 m long synthetic indoor running track (Rekortan, Sport Group, Burgheim, Germany) in the Biomechanics Laboratory at the Blacktown Exercise Sports and Technology Hub. All participants wore their own running shoes and shorts for the duration of testing. Prior to each condition, participants were asked to perform a standardised warmup of four to five trials where they incrementally increased their running velocity based on verbal guidance from the research team. The participants then performed forward and backward running bouts at 40%–100% of their maximum velocity; forward running was completed first for all participants. In each running direction, the participants first completed three maximal sprint trials from a staggered standing start. Following this, participants then completed a minimum of three submaximal velocity trials in a controlled order, where participants were guided to achieve running efforts at ∼90%, ∼70% and ∼40% of maximum, ensuring a spread of trials across the velocity spectrum. The runway provided the participants with 40 m to accelerate to and maintain a constant velocity. To help monitor the average running velocity of each trial, timing gates (SmartSpeed, VALD Performance, Brisbane, Queensland, Australia) were positioned at the end of the data collection volume. Participants were permitted self‐selected recovery periods of up to 5 minutes between trials to minimise fatigue.

### 2.3 Instrumentation

Spherical reflective 14 mm markers were attached directly onto each participant's shoes based on underlying bony landmarks of the calcaneus and navicular, and on each participant's sacrum. Three‐dimensional marker trajectories were sampled at 250 Hz using a 22‐camera optoelectronic motion analysis system (Valkyrie and Nexus, Vicon Motion Systems, Oxford Metrics Ltd., Oxford, United Kingdom). Three‐dimensional ground reaction forces were sampled synchronously with the marker data at 1000 Hz with 10 strain gauge force plates embedded in series in the runway (Advanced Mechanical Technology Inc., Watertown, Massachusetts, United States of America). The anterior‐posterior plane of the laboratory was aligned with the long axis of the runway. The capture volume was positioned between the 30 and 40 m points of the runway, to enable the capture of constant velocity running while allowing 20 m to decelerate.

### 2.4 Data Processing

Marker trajectories were labelled in Vicon Nexus (version 2.16, Vicon Motion Systems, Oxford Metrics Ltd., Oxford, United Kingdom), and vertical ground reaction forces were used to identify initial contact and toe‐off with a 20 N threshold. Marker trajectories were low‐pass filtered using a zero‐lag, fourth order Butterworth filter with a cut‐off frequency of 12 Hz.

Running was defined as a repetitive gait cycle consisting of a ground contact phase (from initial contact to toe‐off) and a swing phase (from toe‐off to initial contact), with one stride representing the full cycle of a single leg from initial contact to the next ipsilateral contact. Temporal measures (i.e., stride time, ground contact time, and swing time) were computed by dividing the number of frames between the respective initial contact and toe‐off events by the sample frequency, with stride frequency computed as the reciprocal of stride time. Stride length was defined as the distance travelled by the foot during each full running cycle and was calculated as the displacement in the anterior‐posterior plane of the laboratory from a marker placed on the calcaneus. Ground contact distance and swing distance were defined as the distance travelled by a proxy of the centre of mass during the ground contact phases and the swing phases, respectively, and were calculated as the displacement in the anterior‐posterior plane of the laboratory from a marker placed on the sacrum. We computed step length and step frequency to support comparisons with prior studies. For descriptions of step‐level analyses, please refer to Supporting Information S1: Appendix A.

**FIGURE 1:**
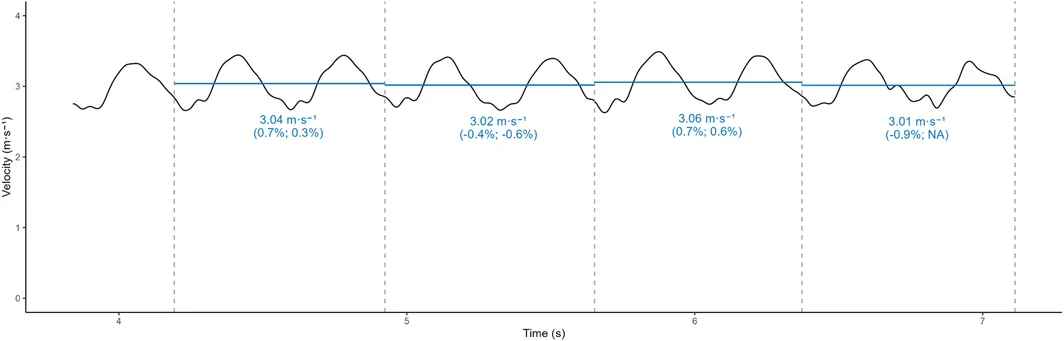
Forward running velocity trace across an example full trial for a participant running at ∼40% of maximum. Vertical dashed lines indicate the foot strike events that define the separate strides on the right leg. The height of the blue horizontal lines indicates the respective right leg stride velocities. Stride velocities are also displayed below each stride; the percentage difference between the previous and current strides, and subsequent and current strides, respectively, are reported in parentheses. NA: indicates that a full stride prior or subsequent to the stride of interest was not available for comparison.

Stride velocity measures were derived from a marker placed on the sacrum and computed by dividing the marker displacement in the anterior‐posterior plane of the laboratory by the stride time. To ensure that only constant‐velocity running was analysed, strides were retained only if their velocity differed by less than 5% relative to the preceding and subsequent strides. This threshold excluded accelerations while preserving natural stride‐to‐stride variability (Figure 1). After applying this criterion, 922 strides were included in the final analyses. Stride velocity was then expressed in two forms: (1) absolute velocity (m·s^−1^) and (2) relative velocity, calculated as the percentage of each participant's individual maximal stride velocity (%*V*_max_). Maximal velocity was taken as the highest stride‐averaged marker‐derived velocity achieved across the three maximal sprint trials, using only those strides that met the criteria for constant‐velocity running.

### 2.5 Statistical Analysis

Relationships between running velocity and spatiotemporal variables were modelled using mixed effects segmented regression with breakpoint estimation in R (R Version 4.5.0, RStudio Version 2025.05.1 + 513, Posit Software, Boston, Massachusetts, United States of America). Breakpoints were estimated using a segmented regression algorithm (*segmented.lme* (V. Muggeo 2016)) which iteratively estimated the breakpoint and its confidence interval while accounting for subject‐level random intercepts. These breakpoint estimates were then carried forward into a hinge‐type segmented mixed effects model. In this model, running velocity and a hinge term were included as fixed effects, with subject‐specific random intercepts to account for repeated measures:

$$Y_{ij}=\beta_{0}+\beta_{1}{Velocity}_{ij}+\delta ({Velocity}_{ij}-{\psi )}_{+}+u_{i}+\varepsilon_{ij}$$

With:

$$\left({Velocity}_{ij}-{\psi )}_{+}=m\text{ax}(0,{Velocity}_{ij}-\psi \right)$$

Where *ψ* is the estimated breakpoint velocity.

Running velocity was treated as a continuous absolute variable (m·s^−1^), consistent with prior literature examining velocity‐dependent biomechanics (Willer et al. 2014; Dorn et al. 2012). A likelihood ratio test was conducted to test the significance of the hinge term in the model compared to a non‐hinge type model. Marginal and conditional *R* ^2^ were computed to quantify the variance explained by the fixed effects and by the full mixed‐effects structure. This process was repeated for every variable, producing separate sets of estimated breakpoints for forward and backward running. Additionally, we conducted a secondary analysis in which running velocity was expressed relative to each participant's maximum velocity (%*V*_max_). This followed the same analysis procedure as described above. Model assumptions were assessed using residual diagnostics, including normality, homoscedasticity, and within‐subject independence. The alpha level was set at 0.05. We interpret marginal and conditional *R* ^2^ descriptively (> 0.50 described as “high”), noting that segmented models typically yield high explanatory power when true breakpoints exist (V. M. R. Muggeo 2003). For detailed descriptions of breakpoint estimation and model specification, please refer to Supporting Information S1: Appendix B.

## 3 Results

The final dataset consisted of 922 strides, with running velocities ranging from 3.01 to 8.45 m·s^−1^ in forward running and 0.95–5.31 m·s^−1^ in backward running. Residual diagnostics confirmed that model assumptions were met. Segmented mixed effects models identified distinct velocity‐dependent transitions in all spatiotemporal variables. Results were largely consistent when velocity was reported in absolute and relative terms. Henceforth, our results are presented with velocity reported in absolute terms. To support the interpretation of practical velocity thresholds, findings from our secondary analysis with velocity quantified as a percentage of maximum are presented in brief here and provided in full in the Supporting Information S1: Appendix C Table S3 and Figure S2. Additionally, for findings from our step‐level analysis, please refer to Supporting Information S1: Appendix C Table S2 and Figure S1.

We found a greater reliance on increasing stride length at lower velocities, but a greater reliance on stride frequency as athletes approached maximum velocity. The change in slope at the breakpoint (*δ*) was significant for all variables in both forward and backward running (*p* < 0.001), and models with a hinge term provided a better fit than non‐hinge type models (*p* < 0.001). Marginal *R* ^2^ values were high for nearly all spatiotemporal variables (0.50–0.94; Supporting Information S1: Appendix C Table S2) with small increases in conditional *R* ^2^, indicating strong fixed effect contributions.

### 3.1 Forward Running

**FIGURE 2:**
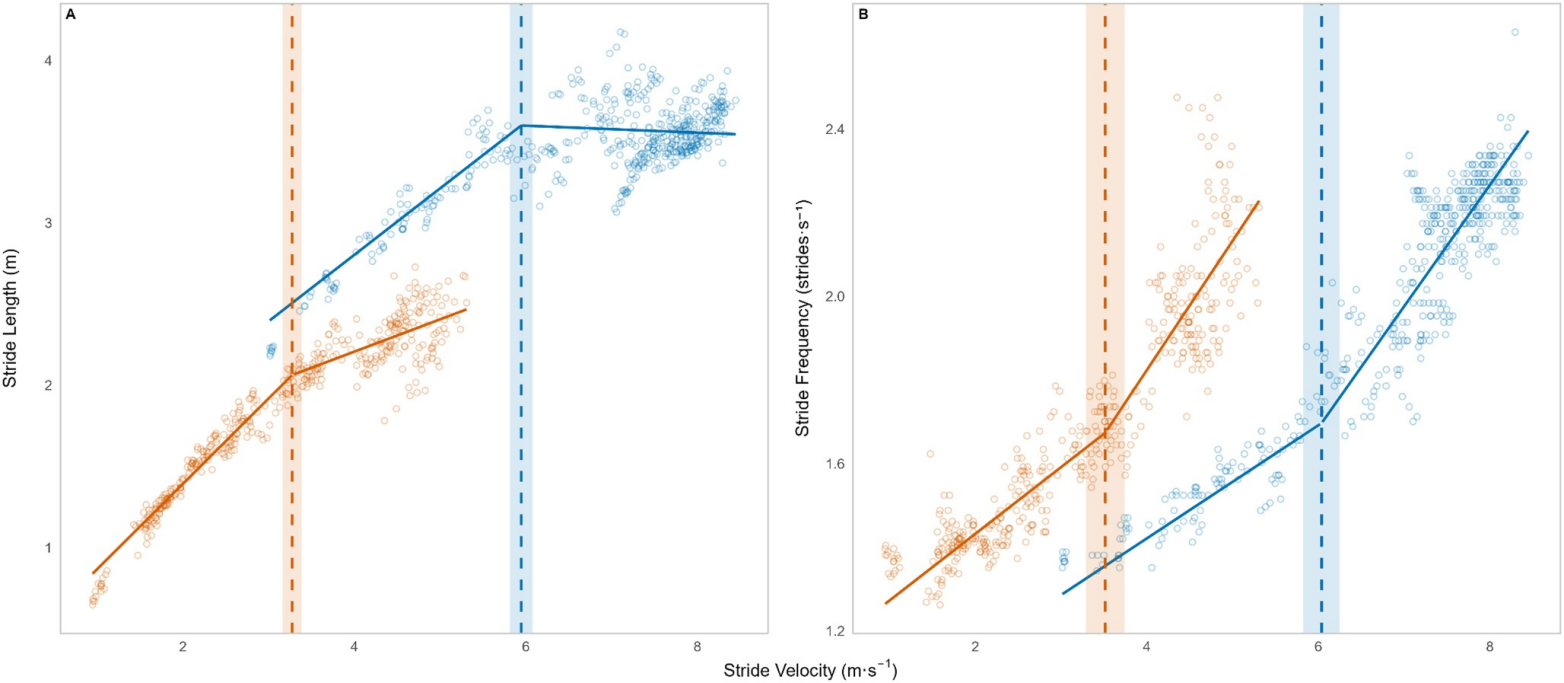
Predicted segmented relationships between forward running (blue) and backward running (orange) velocity and stride length (A), and stride frequency (B). Breakpoints (vertical dashed lines), along with their corresponding 95% confidence intervals (shaded area), indicate distinct transitions in the relationship between spatiotemporal variables and velocity.

Stride length increased steeply with increasing forward running velocity, lengthening by 0.41 m for every 1 m·s^−1^ increase in velocity until reaching a breakpoint at 5.95 m·s^−1^ (95% CI: 5.81–6.08; Figure 2A). Above this velocity, stride length plateaued (−0.02 m per 1 m·s^−1^). Stride frequency increased at a rate of 0.14 strides·s^−1^ for every 1 m·s^−1^ increase in velocity prior to its breakpoint at 6.04 m·s^−1^ (95% CI: 5.82–6.25; Figure 2B), but unlike stride length, a steeper increase was observed at higher velocities (0.29 strides·s^−1^ per 1 m·s^−1^; *δ* = 0.15). Our secondary analysis revealed the same distinct plateau in stride length at velocities above 74%*V*_max_ (95% CI: 72–75), alongside a rapid rise in stride frequency (75%*V*_max_; 95% CI: 73–77).

**FIGURE 3:**
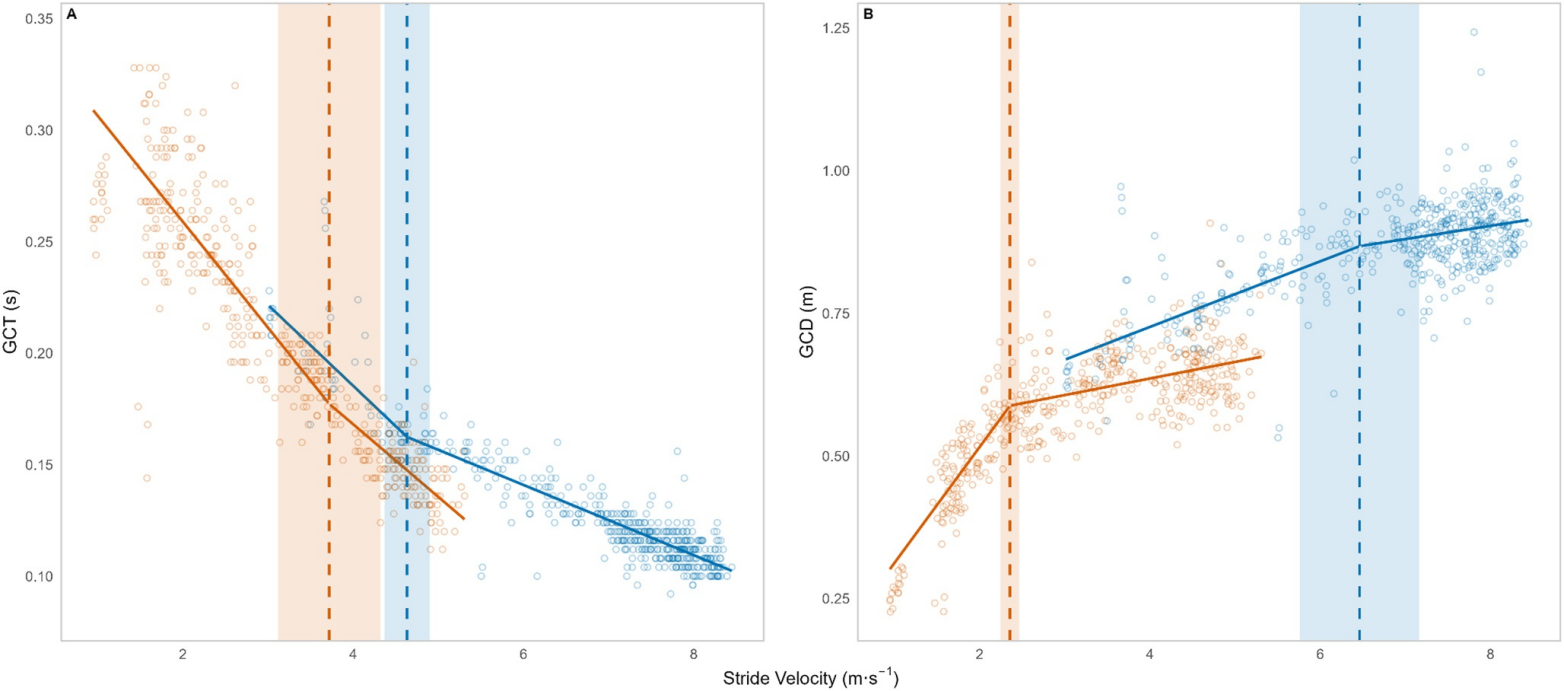
Predicted segmented relationships between forward running (blue) and backward running (orange) velocity and ground contact time (A), and ground contact distance (B). Breakpoints (vertical dashed lines), along with their corresponding 95% confidence intervals (shaded area), indicate distinct transitions in the relationship between spatiotemporal variables and velocity. GCD, Ground contact distance; GCT, Ground contact time.

At velocities under 4.63 m·s^−1^ (95% CI: 4.37–4.90; Figure 3A), ground contact times decreased by −0.04 s for every 1 m·s^−1^ increase in velocity. Above this breakpoint, ground contact time continued to shorten, but by −0.02 s; a slightly more conservative rate than that observed below the breakpoint (*δ* = 0.02). Conversely, ground contact distance increased progressively by 0.06 m for every 1 m·s^−1^ increase in velocity, until a breakpoint at 6.46 m·s^−1^ (95% CI: 5.76–7.17; Figure 3B). Above this breakpoint, like ground contact time, ground contact distance showed a more conversative increase (0.02 m per 1 m·s^−1^; *δ* = −0.03).

**FIGURE 4:**
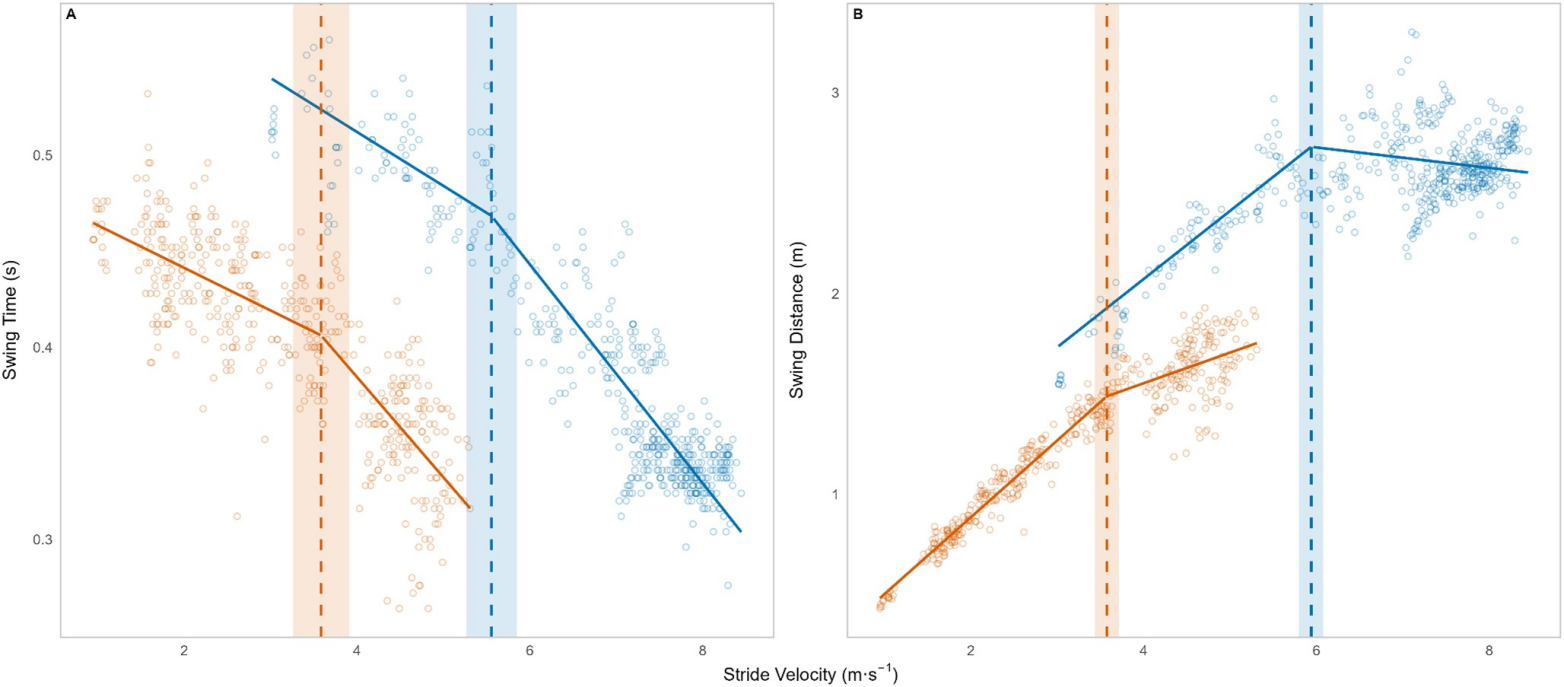
Predicted segmented relationships between forward running (blue) and backward running (orange) velocity and swing time (A), and swing distance (B). Breakpoints (vertical dashed lines), along with their corresponding 95% confidence intervals (shaded area), indicate distinct transitions in the relationship between spatiotemporal variables and velocity.

Swing time decreased gradually, reducing by −0.03 s for every 1 m·s^−1^ increase in velocity, until reaching a breakpoint at 5.56 m·s^−1^ (95% CI: 5.26–5.85; Figure 4A). Above this velocity, swing time exhibited a pronounced reduction (−0.06 s per 1 m·s^−1^; *δ* = −0.03). Swing distance first increased steeply (0.34 m per 1 m·s^−1^) to a peak at 5.94 m·s^−1^ (95% CI: 5.80–6.08; Figure 4B), before decreasing by −0.05 m for every 1 m·s^−1^ increase at higher velocities.

### 3.2 Backward Running

In backward running, a breakpoint in stride length occurred at 3.27 m·s^−1^ (95% CI: 3.16–3.38; Figure 2A). At lower velocities, stride length increased steeply, lengthening by 0.53 m for every 1 m·s^−1^ increase in velocity. Backward running exhibited a strategy distinct from forward running at higher velocities, with stride length displaying a continual but more conservative increase (0.20 m per 1 m·s^−1^; *δ* = −0.33). Stride frequency displayed a similar pattern as in forward running. It increased progressively by 0.16 strides·s^−1^ for every 1 m·s^−1^ increase in velocity, then became steeper beyond the breakpoint (0.31 strides·s^−1^ per 1 m·s^−1^; *δ* = 0.15), which occurred at 3.51 m·s^−1^ (95% CI: 3.29–3.74; Figure 2B). Our secondary analysis of relative velocity supports these findings. The increase in stride length became more conservative at velocities above 66%*V*_max_ (95% CI: 63–68), whereas the increase in stride frequency became more prominent above 67%*V*_max_ (95% CI: 61–73).

Ground contact times decreased progressively as backward running velocity increased, reducing by −0.05 s for every 1 m·s^−1^ increase in velocity, until a breakpoint at 3.72 m·s^−1^ (95% CI: 3.12–4.32; Figure 3A). Above this velocity, ground contact time continued to shorten, but by a slightly more conservative rate of −0.03 s (*δ* = 0.01). In contrast, at velocities under 2.36 m·s^−1^ (95% CI: 2.25–2.47; Figure 3B), ground contact distance increased steeply by 0.20 m for every 1 m·s^−1^ increase in velocity. Beyond this velocity, the increase in ground contact distance became markedly more conservative, increasing by just 0.03 m for every 1 m·s^−1^ increase in velocity (*δ* = −0.17).

Swing time decreased by −0.02 s for every 1 m·s^−1^ increase in velocity prior to its breakpoint at 3.58 m·s^−1^ (95% CI: 3.26–3.91; Figure 4A) but showed a steeper increase at higher velocities (−0.05 s per 1 m·s^−1^; *δ* = −0.03). Swing distance increased steeply with velocity, lengthening by 0.38 m for every 1 m·s^−1^ increase in velocity prior to its breakpoint, which occurred at 3.57 m·s^−1^ (95% CI: 3.43–3.71; Figure 4B). Above this velocity, swing distance continued to rise, but more conservatively than before its breakpoint (0.15 m per 1 m·s^−1^; *δ* = −0.23).

## 4 Discussion

The purpose of this study was to (I) examine the relationship between spatiotemporal variables and velocity in forward and backward running, and (II) identify distinct transitions in this relationship. We have identified distinct velocity‐dependent transitions, whereby the spatiotemporal strategy that female rugby sevens athletes use in forward and backward running differs when running at lower or higher velocities. At lower velocities, we observed a greater reliance on increasing stride length, but its contribution declined as velocity increased. Stride frequency showed a different pattern, with a prominent increase as athletes approached maximum forward and backward running velocities. Consequently, stride frequency became the primary mechanical driver at top speeds. Backward running displayed some spatiotemporal characteristics that diverge from those seen in forward running, suggesting that increases in velocity are uniquely generated and constrained when running backward; highlighting direction‐specific training targets for forward and backward running. This information allows practitioners to develop training interventions that adequately target the mechanical drivers of performance in forward and backward running.

### 4.1 Forward Running

At lower velocities, forward running was characterised by increases in stride length alongside simultaneous increases in stride frequency. Although our findings show that both variables contribute to improvements in velocity at 3.01–5.95 m·s^−1^, previous work consistently shows that relative increases in stride length are much greater at slower speeds (Willer et al. 2014; Dorn et al. 2012; Schache et al. 2014), indicating that it is the dominant driver of performance at lower velocities. Our findings support a greater reliance on increasing stride length at < 5.95 m·s^−1^ for two reasons. First, longer ground contact times create a mechanical environment that facilitates longer strides by allowing more time to generate the upward and forward impulse that drives centre of mass displacement (Willer et al. 2014; Dorn et al. 2012). Second, we observed substantial increases in ground contact distance and swing distance at lower velocities. When the centre of mass travels further upward and forward, this effectively increases the spatial range within which the leg can be repositioned (Hunter et al. 2004). Taken together, these findings suggest a strong link between stride length, centre of mass displacement, and a higher impulse during ground contact. Indeed, the ability to apply large forces to the ground during ever‐decreasing periods of ground contact has been shown to be the greatest indicator of running performance (Weyand et al. 2010). Athletes should therefore look to maximise propulsive forces during ground contact, as this contributes strongly to performance, particularly at speeds below ∼75%*V*_max_.

At velocities above 5.95 m·s^−1^ in forward running, stride length plateaued. Our findings are consistent with observations in sprint and distance runners who display the same distinct shift at similar relative velocities (Willer et al. 2014; Dorn et al. 2012; Weyand et al. 2000). At these higher velocities, short ground contact times reduce the time available for force production. As the ankle plantar flexors are primarily responsible for increasing stride length, their diminishing capacity to generate peak forces and perform positive work under shorter ground contact times likely contributes to the plateau (Willer et al. 2014; Dorn et al. 2012). This plateau coincided with decreases in swing distance, indicating that aerial contributions to stride length diminish as velocity increases, as the leg has less spatial range to progress forward. Ground contact distance continued to increase with increasing velocity, but this was modest and insufficient to offset the reductions in aerial displacement. Consequently, athletes must shift to increasing stride frequency to continue increasing forward running velocity. This transition is reflected in the steeper post‐breakpoint slopes for stride frequency, and the pronounced reduction in swing time at higher velocities. Stride frequency depends on substantial reductions in swing time to facilitate faster limb cycling (Willer et al. 2014; Weyand et al. 2000). Training should therefore promote rapid acceleration and repositioning of the leg during swing (e.g., via overspeed and resisted running), thereby increasing the frequency at which athletes push on the ground. These strategies align with the known role of the hip musculature in driving hip‐ and knee‐joint accelerations during high‐speed forward running (Willer et al. 2014; Dorn et al. 2012). Thus, consistent with previous recommendations (Schache et al. 2014), enhancing hip muscle force production may well present an effective strategy for improving forward running performance, particularly at speeds above ∼75%*V*_max_.

### 4.2 Backward Running

At lower backward running velocities, simultaneous increases in stride length and stride frequency were observed. The steep increases in ground contact distance and swing distance below their respective breakpoints suggest that backward running, like forward running, initially relies on extending stride length to increase velocity. As velocity increased beyond ∼65%*V*_max_, however, backward running showed a distinct strategy, characterised by a continued increase in stride length. With the absence of heel strike and reduced knee flexion, the braking demands of backward running are minimal (Flynn and Soutas‐Little 1993; Threlkeld et al. 1989; Uthoff et al. 2018). The substantial reduction in horizontal braking likely allows a greater proportion of the stance‐phase to be spent contributing to upward and backward impulse and, thus, centre of mass motion. Additionally, as backward running operates at much lower absolute velocities than forward running, ground contact times remain relatively long and do not constrain the time available for force production. Consequently, stride length, ground contact distance, and swing distance continue to increase, albeit at a more conservative rate than at lower velocities. At a similar velocity, stride frequency began to rise more steeply, accompanied by rapid reductions in swing time. This pattern suggests that high‐speed backward running, like forward running, increasingly depends on the ability to accelerate and reposition the legs quickly, even while attaining longer strides.

Interestingly, visual inspection of the data suggests that stride frequency began to increase more markedly at approximately 1.65 strides·s^−1^ in both forward and backward running. The similarity of this transition point suggests that the shift towards a greater reliance of stride frequency occurs at a comparable stage in both locomotor modes. Furthermore, peak stride frequencies were broadly similar between running directions. Thus, the lower maximal velocities achieved during backward running are unlikely to be explained by limitations in stride frequency. Instead, differences in stride length and the underlying biomechanical factors that determine it are likely to play a more important role. Given the observed direction‐specific characteristics of backward running, athletes may benefit from targeted training aimed at improving backward running velocity, including a focus on both force application during ground contact and rapid leg turnover during the swing‐phase. These findings may have practical implications for athletes in field sports in general, particularly given that backward locomotion is frequently performed by soccer players (Mohr et al. 2003) and referees (Krustrup et al. 2009), and is commonly incorporated into warm‐up and injury prevention programs (Uthoff et al. 2018).

An important consideration when developing training strategies to enhance running performance is the muscular strategy underlying these spatiotemporal patterns. In forward running, the quadriceps contribute primarily to braking, acting eccentrically to control knee flexion and absorb impact during stance, while the ankle plantar flexors and the hip flexors and hamstrings are the dominant contributors to stride length and stride frequency, respectively (Flynn and Soutas‐Little 1993; Hamner et al. 2010). In backward running, however, the role of these muscles is suggested to switch, with the quadriceps instead acting isometrically and concentrically to propel the body backward (Flynn and Soutas‐Little 1993). The findings of Devita & Stribling (Devita and Stribling 1991) support this notion with their discovery that the knee extensors provide the primary propulsive work in backward running, while propulsion in forward running comes from the ankle plantar flexors (Hamner and Delp 2013). Importantly, the present study demonstrated that both stride length and stride frequency increase as backward running velocity increases, indicating that high‐speed backward running is likely not driven by a single spatiotemporal parameter, but by coordinated changes in both stride length and stride frequency. It remains unknown, however, which muscles directly drive stride length and stride frequency in backward running, or whether muscle contributions shift across different velocities. Understanding these relationships represents an important direction for future research, to inform directed targeting of interventions towards the muscles that drive backward running performance. Taken together, it seems that backward running is likely distinct from forward running, in that it relies on actions of the quadriceps for propulsion, rather than the ankle plantar flexors, hip flexors, and hamstrings that drive forward running. This suggests that enhancing force production of the quadriceps may present an effective strategy for improving backward running performance.

### 4.3 Limitations

Our data pertain to controlled laboratory straight‐line running, which is not necessarily reflective of on‐field performance as there is likely greater variability in‐game due to sport‐specific constraints. Understanding these mechanical behaviours in a controlled environment highlights likely mechanisms that could be explored in game or simulated game environments. Additionally, participants did not perform running bouts at set absolute velocities, and instead we obtained experimental gait data across a spread of relative velocities. This was partially a result of set absolute velocities being difficult to attain during overground running. Nevertheless, when combined with modelling running velocity as a continuous fixed effect, this approach has enabled us to identify the velocities at which running mechanics change with much greater precision. Whilst this may make comparisons against other studies more difficult, our findings based on overground running are arguably more reflective of in‐game demands and provide practical training targets. Despite this limitation, average spatiotemporal running data across binned velocities were provided to support these comparisons (Supporting Information S1: Appendix C Table S1). Finally, our data apply to constant‐velocity running and, thus, may not reflect the spatiotemporal strategies used during accelerative and decelerative running. Nevertheless, training targets to improve running performance, such as increasing the application of force during ground contact and limb turnover during the swing‐phases are likely transferable to accelerative running (Nagahara et al. 2014).

## 5 Conclusions

We have identified distinct velocity‐dependent transitions in the running strategy used by female rugby sevens athletes; the velocities at which these transitions occur appear to be largely consistent across spatiotemporal parameters. Forward running velocity appears to increase primarily due to increased stride length at lower velocities, and increased stride frequency at higher velocities. In backward running, whilst similar to forward running at lower velocities, stride length also appears to play an important role in driving top speed, alongside stride frequency. As expected, this transition reflected coordinated changes in ground contact and swing phase mechanics. These distinct velocity‐dependent transitions have extensive implications for interventions to enhance athletic performance. To leverage the primary contributors to forward running performance, we recommend a focus on training interventions to encourage rapid leg turnover during the swing‐phases. Conversely, in backward running, a focus on both rapid application of force during ground contact and rapid leg turnover during the swing‐phases would appear to best leverage the mechanics that differentiate performance at, and approaching, maximal velocity.

## Author Contributions

All authors have made substantial contributions to all aspects of the work and meet the International Committee of Medical Journal Editors (ICMJE) criteria on authorship. All authors agree to the final submitted version and agree to be accountable for all aspects of the work.

## Funding

This research was supported by the Commonwealth through an Australian Government Research Training Programme Scholarship [DOI: https://doi.org/10.82133/C42F‐K220]. The funding organisation has no role in the collection, analysis, and interpretation of data, and in the right to approve or disapprove publication of the finished manuscript.

## Ethics Statement

All procedures were performed in compliance with relevant laws and institutional guidelines and have been approved by the Australian Catholic University Human Research Ethics Committee (Ethics number: 2023‐3814H).

## Consent

Informed consent was obtained from all participants prior to data collection. The privacy rights of participants have been observed.

## Conflicts of Interest

The authors declare no conflicts of interest.

## Supporting information


Supporting Information S1

## Data Availability

The data that supports the findings of this study are available in the supplementary material of this article. Raw research data are not shared.
