# Supplementary material for: Velocity‐Dependent Transitions in Forward and Backward Running Mechanics in Female International Rugby Players
Source: Eur J Sport Sci. 2026 Sep 11;26(10):e70255. doi: 10.1002/ejsc.70255 (PMC13569467; doi:10.1002/ejsc.70255)
Supplement: Supplementary file 1 [file EJSC-26-e70255-s001.pdf]

Supplementary Material

Appendix A: Spatiotemporal computation

We computed various spatiotemporal variables from marker-based optoelectronic motion analysis data. In our primary analyses, running was defined as a repetitive gait cycle, with one stride representing the full cycle of a single leg from initial contact to the next ipsilateral contact. To support comparisons with literature examining step biomechanics, we computed step length and step frequency. One step represents half a running cycle from initial contact to the next contact of the contralateral leg. Step length was calculated as the displacement in the anterior-posterior plane of the laboratory from a marker placed on the calcaneus. Step frequency was computed as the reciprocal of step time, with step time derived by dividing the number of frames between the initial contact events by the sample frequency. For findings from our step-level analyses, please refer to Appendix C of this Supplementary Material.

Appendix B: Model specification

We modelled the association between running velocity and each outcome using a segmented mixed effects regression model (SMM) to detect structural changes in the velocity–outcome relationship. Let Y_{ij} denote the outcome measured for subject $i = 1, 2, \dots, n$ at observation $j = 1, 2, \dots, n_i$, and let $Velocity_{ij}$ denote the corresponding velocity value. Our primary goal is to detect unknown breakpoints in the association between Y_{ij} and $Velocity_{ij}$. Because each subject contributes repeated observations, we specified a mixed-effects structure with a subject-specific random intercept. As an initial step, we fit a baseline linear mixed effects model of the form

$$Y_{ij} = \beta_0 + \beta_1 Velocity_{ij} + u_i + \varepsilon_{ij}, \quad (1)$$

Where $u_i \sim \mathcal{N}(0, \sigma_u^2)$ captures between subject heterogeneity and $\varepsilon_{ij} \sim \mathcal{N}(0, \sigma^2)$ are independent residual errors. In R (R Version 4.5.0, RStudio Version 2025.05.1+513, Posit Software, Boston, Massachusetts, United Stated of America) this model was fit using the *nlme* package via

```r
m0 <- lme(
  fixed     = outcome ~ running_velocity,
  random    = list(subject = ~ 1),
  data      = dat,
  method    = "ML"
)
```

To allow a change in slope at an unknown velocity, we extended this model using the SMM algorithm of Muggeo [1]. Let ψ denote the breakpoint. The SMM augments the baseline model with a hinge-type term, yielding

$$Y_{ij} = \beta_0 + \beta_1 Velocity_{ij} + \delta (Velocity_{ij} - \psi)_+ + u_i + \varepsilon_{ij} \quad (2)$$

In this equation, the hinge function is defined as $(Velocity_{ij} - \psi)_+ = \max(0, Velocity_{ij} - \psi)$. The model assumes that the relationship between Y_{ij} and $Velocity_{ij}$ follows two linear regimes: (1) when $Velocity_{ij} \leq \psi$, the hinge term is zero, and the slope is simply β_1; (2) when $Velocity_{ij} > \psi$, the hinge term becomes $Velocity_{ij} - \psi$, and the slope becomes $\beta_1 + \delta$. Thus, β_1 is the slope of the trajectory before the breakpoints, δ is the change in slope after the breakpoint, and $\beta_1 + \delta$ is the slope after the breakpoint. We estimated the unknown breakpoints, along with their corresponding 95% confidence intervals (CIs). Breakpoint estimation was initialised at the median observed velocity and obtained using the *segmented* package via

```r
seg <- segmented.lme(
  obj   = m0,
  seg.Z = ~ running_velocity,
  psi   = median(dat$running_velocity),
  npsi  = 1
)
```

These breakpoint estimates $\hat{\psi}$ were then treated as fixed hinge locations in a subsequent hinge-type mixed effects model. For each outcome–task combination, we constructed the hinge term

```
dat$hinge <- pmax(0, dat$running_velocity - bp)
```

and fit the hinge model

```
  m1 <- lme(
    fixed  = outcome ~ running_velocity + hinge,
    random = ~ 1 | Subject,
    data   = dat,
    method = "ML"
  )
```

Corresponding to equation (2) with $\psi = \hat{\psi}$. The fixed effect coefficients yield the pre-breakpoint slope β_1, the slope change δ, and the post-breakpoint slope $\beta_1 + \delta$. The full parameter set for the segmented model is $\Phi = (\psi)$ and $\Theta = (\beta_0, \beta_1, \delta, \sigma_u^2, \sigma^2)$, with ψ treated as a known in the hinge-type model. All parameters were estimated via maximum likelihood.

Appendix C: Supplemental results

Table S1. Average spatiotemporal running data across binned velocities.

Variable	Backward running						Forward running					
	<1 m·s^{-1}	1-1.99 m·s^{-1}	2-2.99 m·s^{-1}	3-3.99 m·s^{-1}	4-4.99 m·s^{-1}	5-5.99 m·s^{-1}	3-3.99 m·s^{-1}	4-4.99 m·s^{-1}	5-5.99 m·s^{-1}	6-6.99 m·s^{-1}	7-7.99 m·s^{-1}	8-8.99 m·s^{-1}
Stride length (m)	0.68 ± 0.03	1.19 ± 0.17	1.66 ± 0.13	2.09 ± 0.13	2.30 ± 0.17	2.47 ± 0.13	2.48 ± 0.19	3.02 ± 0.12	3.40 ± 0.15	3.57 ± 0.20	3.53 ± 0.18	3.65 ± 0.12
Stride frequency (strides·s^{-1})	1.38 ± 0.02	1.40 ± 0.06	1.49 ± 0.09	1.67 ± 0.06	2.01 ± 0.16	2.12 ± 0.10	1.40 ± 0.04	1.51 ± 0.06	1.64 ± 0.08	1.86 ± 0.09	2.16 ± 0.13	2.27 ± 0.08
Step length (m)	0.35 ± 0.07	0.59 ± 0.09	0.83 ± 0.07	1.04 ± 0.07	1.13 ± 0.09	1.21 ± 0.08	1.24 ± 0.10	1.50 ± 0.06	1.69 ± 0.08	1.78 ± 0.11	1.75 ± 0.10	1.82 ± 0.06
Step frequency (steps·s^{-1})	2.80 ± 0.06	2.80 ± 0.17	3.00 ± 0.25	3.35 ± 0.19	4.04 ± 0.40	4.24 ± 0.30	2.81 ± 0.29	3.03 ± 0.14	3.29 ± 0.19	3.71 ± 0.21	4.33 ± 0.29	4.53 ± 0.20
GCT (s)	0.26 ± 0.01	0.27 ± 0.03	0.24 ± 0.03	0.19 ± 0.01	0.15 ± 0.01	0.14 ± 0.01	0.21 ± 0.03	0.17 ± 0.01	0.15 ± 0.01	0.13 ± 0.01	0.12 ± 0.01	0.11 ± 0.01
GCD (m)	0.25 ± 0.02	0.44 ± 0.08	0.58 ± 0.06	0.64 ± 0.04	0.65 ± 0.06	0.66 ± 0.06	0.71 ± 0.10	0.76 ± 0.05	0.82 ± 0.08	0.87 ± 0.06	0.89 ± 0.06	0.91 ± 0.05
Swing time (s)	0.46 ± 0.01	0.45 ± 0.03	0.43 ± 0.03	0.41 ± 0.02	0.35 ± 0.03	0.34 ± 0.02	0.51 ± 0.03	0.49 ± 0.02	0.46 ± 0.03	0.41 ± 0.02	0.35 ± 0.03	0.33 ± 0.01
Swing distance (m)	0.45 ± 0.02	0.75 ± 0.11	1.08 ± 0.11	1.44 ± 0.11	1.62 ± 0.15	1.77 ± 0.10	1.77 ± 0.16	2.26 ± 0.11	2.56 ± 0.15	2.67 ± 0.17	2.62 ± 0.17	2.71 ± 0.13

Data are presented as mean ± standard deviation. GCT: Ground contact time; GCD: Ground contact distance.

Table S2. Hinge-type mixed effects model results for the absolute velocity (m·s⁻¹) analysis.

Task	Variable	Breakpoint [95% CI]	β_1 [95% CI]	$\beta_1 + \delta$ [95% CI]	Hinge [95% CI]	Hinge p	Marginal R^2	Conditional R^2	LRT p
Forward running	Stride length (m)	5.95 [5.81, 6.08]	0.41 [0.39, 0.43]	-0.02 [-0.06, 0.02]	-0.43 [-0.46, -0.40]	<.001	0.70	0.87	<.001
	Stride frequency (strides·s⁻¹)	6.04 [5.82, 6.25]	0.14 [0.12, 0.15]	0.29 [0.27, 0.31]	0.15 [0.13, 0.18]	<.001	0.90	0.95	<.001
	Step length (m)	5.96 [5.80, 6.11]	0.20 [0.19, 0.21]	-0.01 [-0.03, 0.01]	-0.21 [-0.23, -0.19]	<.001	0.67	0.83	<.001
	Step frequency (steps·s⁻¹)	6.05 [5.75, 6.34]	0.27 [0.23, 0.30]	0.58 [0.52, 0.65]	0.31 [0.26, 0.37]	<.001	0.86	0.91	<.001
	GCT (s)	4.63 [4.37, 4.90]	-0.04 [-0.04, -0.03]	-0.02 [-0.02, -0.01]	0.02 [0.02, 0.02]	<.001	0.86	0.88	<.001
	GCD (m)	6.46 [5.76, 7.17]	0.06 [0.05, 0.07]	0.02 [0.005, 0.04]	-0.03 [-0.05, -0.02]	<.001	0.50	0.58	<.001
	Swing time (s)	5.56 [5.26, 5.85]	-0.03 [-0.03, -0.02]	-0.06 [-0.06, -0.05]	-0.03 [-0.03, -0.02]	<.001	0.88	0.95	<.001
	Swing distance (m)	5.94 [5.80, 6.08]	0.34 [0.32, 0.36]	-0.05 [-0.09, -0.01]	-0.39 [-0.42, -0.36]	<.001	0.60	0.82	<.001

Task	Variable	Breakpoint [95% CI]	β_1 [95% CI]	$\beta_1 + \delta$ [95% CI]	Hinge [95% CI]	Hinge p	Marginal R^2	Conditional R^2	LRT p
Backward running	Stride length (m)	3.27 [3.16, 3.38]	0.53 [0.51, 0.54]	0.20 [0.16, 0.23]	-0.33 [-0.36, -0.30]	<.001	0.94	0.97	<.001
	Stride frequency (strides·s⁻¹)	3.51 [3.29, 3.74]	0.16 [0.15, 0.17]	0.31 [0.28, 0.34]	0.15 [0.12, 0.18]	<.001	0.87	0.93	<.001
	Step length (m)	3.24 [3.11, 3.37]	0.26 [0.25, 0.27]	0.09 [0.07, 0.11]	-0.17 [-0.19, -0.15]	<.001	0.92	0.95	<.001
	Step frequency (steps·s⁻¹)	3.47 [3.11, 3.83]	0.33[0.28, 0.37]	0.60 [0.51, 0.70]	0.28 [0.19, 0.37]	<.001	0.79	0.84	<.001
	GCT (s)	3.72 [3.12, 4.32]	-0.05 [-0.05, -0.04]	-0.03 [-0.04, -0.02]	0.01 [0.01, 0.02]	<.001	0.86	0.90	<.001
	GCD (m)	2.36 [2.25, 2.47]	0.20 [0.18, 0.22]	0.03 [0.0003, 0.06]	-0.17 [-0.20, -0.15]	<.001	0.72	0.80	<.001
	Swing time (s)	3.58 [3.26, 3.91]	-0.02 [-0.03, -0.02]	-0.05 [-0.06, -0.04]	-0.03 [-0.04, -0.02]	<.001	0.68	0.80	<.001
	Swing distance (m)	3.57 [3.43, 3.71]	0.38 [0.37, 0.39]	0.15 [0.12, 0.18]	-0.23 [-0.26, -0.20]	<.001	0.93	0.96	<.001

GCT: Ground contact time; GCD: Ground contact distance; LRT: Likelihood ratio test; β_1: slope before the breakpoint; $\beta_1 + \delta$: slope after the breakpoint; δ: change in slope.

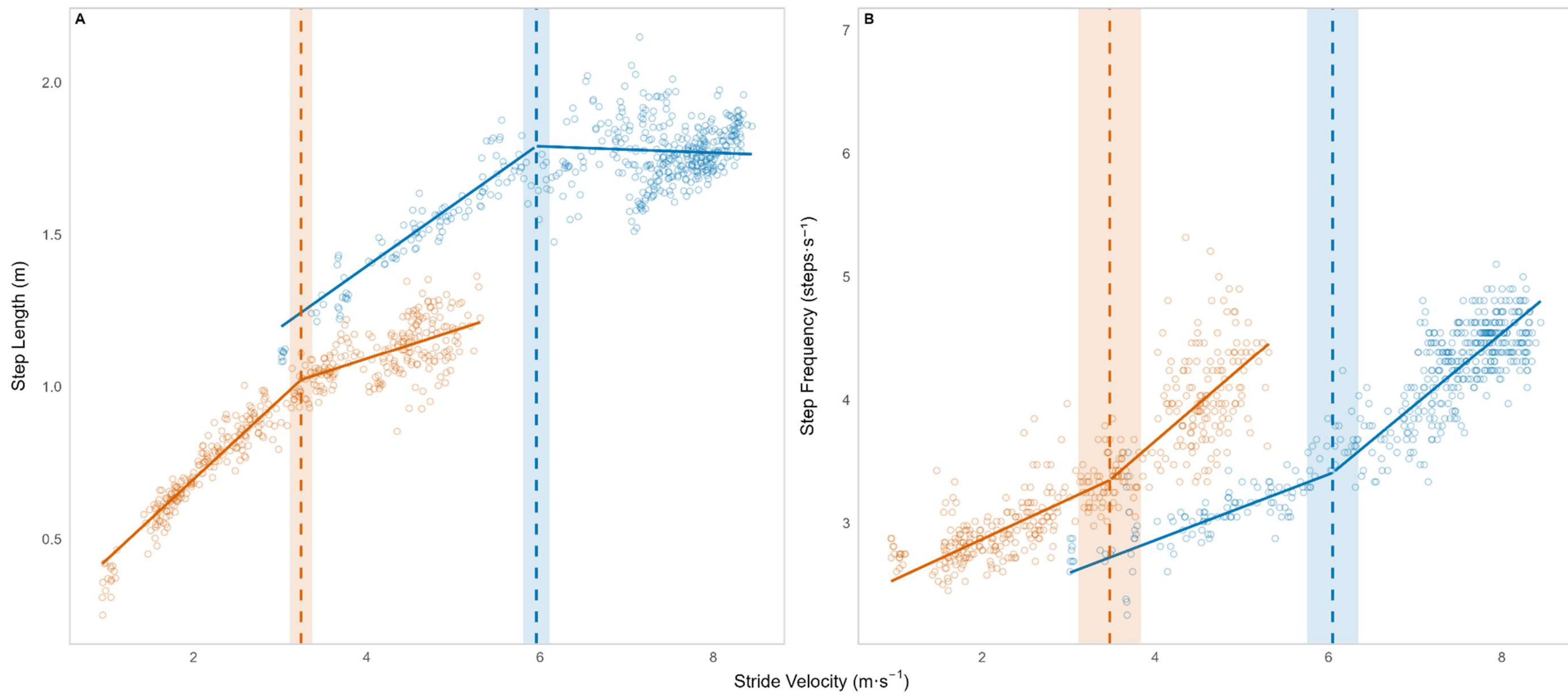

Figure S1. Predicted segmented relationships between forward running (blue) and backward running (orange) velocity and step length (A) and step frequency (B). Breakpoints (vertical dashed lines), along with their corresponding 95% confidence intervals (shaded area), indicate distinct transitions in the relationship between spatiotemporal variables and velocity.

Table S3. Hinge-type mixed effects model results for the relative velocity (%V_{max}) analysis.

Task	Outcome	Breakpoint [95% CI]	β_1 [95% CI]	$\beta_1 + \delta$ [95% CI]	Hinge [95% CI]	Hinge p	Marginal R^2	Conditional R^2	LRT p
Forward running	Stride length (m)	73.90 [72.44, 75.35]	0.04 [0.03, 0.04]	-0.003 [-0.01, 0.0002]	-0.04 [-0.04, -0.04]	<.001	0.70	0.89	<.001
	Stride frequency (strides·s⁻¹)	75.16 [73.01, 77.31]	0.01 [0.01, 0.01]	0.02 [0.02, 0.03]	0.02 [0.01, 0.02]	<.001	0.93	0.95	<.001
	Step length (m)	74.24 [72.50, 75.97]	0.02 [0.02, 0.02]	-0.002 [-0.004, 0.0003]	-0.02 [-0.02, -0.02]	<.001	0.67	0.86	<.001
	Step frequency (steps·s⁻¹)	75.27 [72.20, 78.34]	0.02 [0.01, 0.02]	0.05 [0.04, 0.05]	0.03 [0.03, 0.04]	<.001	0.89	0.91	<.001
	GCT (s)	62.93 [58.04, 67.82]	-0.002 [-0.003, -0.002]	-0.001 [-0.002, -0.001]	0.001 [0.001, 0.002]	<.001	0.83	0.87	<.001
	GCD (m)	76.47 [67.69, 85.24]	0.01 [0.004, 0.01]	0.002 [0.0004, 0.004]	-0.003 [-0.005, -0.002]	<.001	0.48	0.58	<.001
	Swing time (s)	67.74 [65.09, 70.38]	-0.001 [-0.002, -0.001]	-0.005 [-0.01, -0.004]	-0.003 [-0.004, -0.003]	<.001	0.92	0.94	<.001
	Swing distance (m)	71.94 [70.24, 73.63]	0.03 [0.03, 0.03]	-0.004 [-0.01, -0.0004]	-0.04 [-0.04, -0.03]	<.001	0.61	0.83	<.001

Task	Outcome	Breakpoint [95% CI]	β_1 [95% CI]	$\beta_1 + \delta$ [95% CI]	Hinge [95% CI]	Hinge p	Marginal R^2	Conditional R^2	LRT p
Backward running	Stride length (m)	65.71 [63.12, 68.30]	0.03 [0.02, 0.03]	0.01 [0.01, 0.01]	-0.01 [-0.02, -0.01]	<.001	0.90	0.97	<.001
	Stride frequency (strides·s⁻¹)	66.75 [60.74, 72.75]	0.01 [0.01, 0.01]	0.01 [0.01, 0.02]	0.01 [0.005, 0.01]	<.001	0.81	0.91	<.001
	Step length (m)	65.87 [62.77, 68.98]	0.01 [0.01, 0.01]	0.005 [0.004, 0.01]	-0.01 [-0.01, -0.01]	<.001	0.88	0.95	<.001
	Step frequency (steps·s⁻¹)	68.14 [59.42, 76.86]	0.01 [0.01, 0.02]	0.03 [0.02, 0.03]	0.01 [0.01, 0.02]	<.001	0.74	0.83	<.001
	GCT (s)	80.27 [69.33, 91.21]	-0.002 [-0.002, -0.002]	-0.001 [-0.002, -0.001]	0.001 [0.001, 0.001]	<.001	0.82	0.90	<.001
	GCD (m)	49.15 [46.63, 51.68]	0.01 [0.01, 0.01]	0.002 [0.0003, 0.003]	-0.01 [-0.01, -0.01]	<.001	0.69	0.79	<.001
	Swing time (s)	69.70 [62.14, 77.26]	-0.001 [-0.001, -0.001]	-0.002 [-0.003, -0.002]	-0.001 [-0.002, -0.001]	<.001	0.65	0.78	<.001
	Swing distance (m)	70.25 [66.80, 73.69]	0.02 [0.02, 0.02]	0.01 [0.01, 0.01]	-0.01 [-0.01, -0.01]	<.001	0.89	0.96	<.001

GCT: Ground contact time; GCD: Ground contact distance; LRT: Likelihood ratio test; β_1: slope before the breakpoint; $\beta_1 + \delta$: slope after the breakpoint; δ: change in slope.

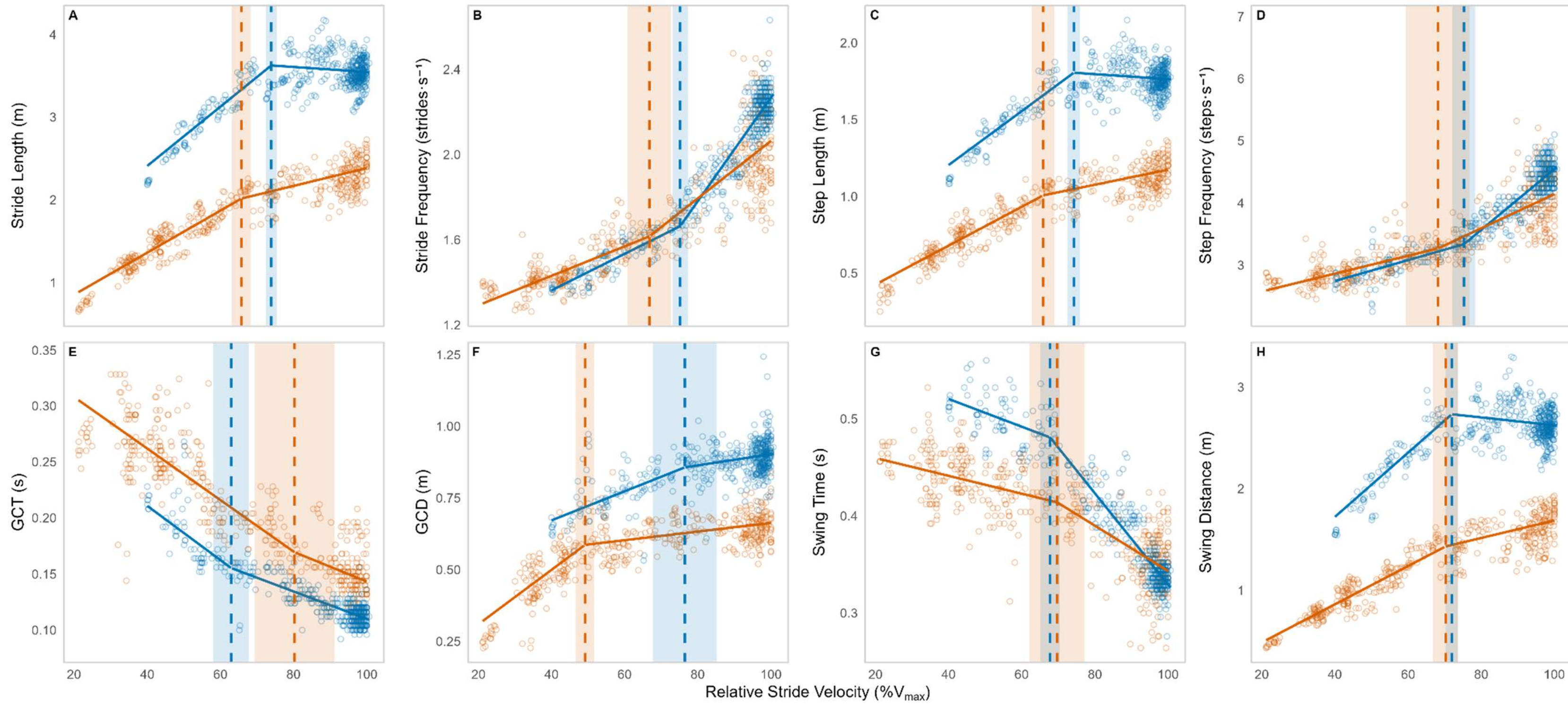

Figure S2. Predicted segmented relationships between relative forward running (blue) and backward running (orange) velocity and stride length (A), stride frequency (B), step length (C), step frequency (D), ground contact time (E), ground contact distance (F), swing time (G), and swing distance (H). Breakpoints (vertical dashed lines), along with their corresponding 95% confidence intervals (shaded area), indicate distinct transitions in the relationship between spatiotemporal variables and velocity. GCT: Ground contact time; GCD: Ground contact distance.

References

1. Muggeo VMR, Atkins DC, Gallop RJ et al. Segmented mixed models with random changepoints: a maximum likelihood approach with application to treatment for depression study. Statistical Modelling 2014; 14(4): 293–313. https://doi.org/10.1177/1471082X13504721
